# Antibiotic and antifungal use in pediatric leukemia and lymphoma patients are associated with increasing opportunistic pathogens and decreasing bacteria responsible for activities that enhance colonic defense

**DOI:** 10.3389/fcimb.2022.924707

**Published:** 2022-07-27

**Authors:** Katherine A. Dunn, Tamara MacDonald, Gloria J. Rodrigues, Zara Forbrigger, Joseph P. Bielawski, Morgan G.I. Langille, Johan Van Limbergen, Ketan Kulkarni

**Affiliations:** ^1^ Department of Pediatrics, Division of Hematology and Oncology, Izaak Walton Killam (IWK) Health, Halifax, NS, Canada; ^2^ Department of Biology, Dalhousie University, Halifax, NS, Canada; ^3^ Institute for Comparative Genomics, Dalhousie University, Halifax, NS, Canada; ^4^ Department of Pharmacy, IWK Health, Halifax, NS, Canada; ^5^ Faculty of Health Professions, Dalhousie University, Halifax, NS, Canada; ^6^ Faculty of Medicine, Dalhousie University, Halifax, NS, Canada; ^7^ Department of Pathology, Dalhousie University, Halifax, NS, Canada; ^8^ Department of Mathematics & Statistics, Dalhousie University, Halifax, NS, Canada; ^9^ Department of Pharmacology, Faculty of Medicine, Dalhousie University, Halifax, NS, Canada; ^10^ Department of Paediatric Gastroenterology and Nutrition, Emma Children’s Hospital, Amsterdam University Medical Centers, Amsterdam, Netherlands; ^11^ Tytgat Institute for Liver and Intestinal Research, Amsterdam Gastroenterology Endocrinology and Metabolism, Amsterdam University Medical Centers, University of Amsterdam, Amsterdam, Netherlands

**Keywords:** leukemia, lymphoma, pediatric, microbiome, antibiotics, antifungals

## Abstract

Due to decreased immunity, both antibiotics and antifungals are regularly used in pediatric hematologic-cancer patients as a means to prevent severe infections and febrile neutropenia. The general effect of antibiotics on the human gut microbiome is profound, yielding decreased diversity and changes in community structure. However, the specific effect on pediatric oncology patients is not well-studied. The effect of antifungal use is even less understood, having been studied only in mouse models. Because the composition of the gut microbiome is associated with regulation of hematopoiesis, immune function and gastrointestinal integrity, changes within the patient gut can have implications for the clinical management of hematologic malignancies. The pediatric population is particularly challenging because the composition of the microbiome is age dependent, with some of the most pronounced changes occurring in the first three years of life. We investigated how antibiotic and antifungal use shapes the taxonomic composition of the stool microbiome in pediatric patients with leukemia and lymphoma, as inferred from both 16S rRNA and metagenome data. Associations with age, antibiotic use and antifungal use were investigated using multiple analysis methods. In addition, multivariable differential abundance was used to identify and assess specific taxa that were associated with multiple variables. Both antibiotics and antifungals were linked to a general decline in diversity in stool samples, which included a decrease in relative abundance in butyrate producers that play a critical role in host gut physiology (*e.g.*, *Faecalibacterium*, *Anaerostipes, Dorea, Blautia*),. Furthermore, antifungal use was associated with a significant increase in relative abundance of opportunistic pathogens. Collectively, these findings have important implications for the treatment of leukemia and lymphoma patients. Butyrate is important for gastrointestinal integrity; it inhibits inflammation, reinforces colonic defense, mucosal immunity. and decreases oxidative stress. The routine use of broad-spectrum anti-infectives in pediatric oncology patients could simultaneously contribute to a decline in gastrointestinal integrity and colonic defense while promoting increases in opportunistic pathogens within the patient gut. Because the gut microbiome has been linked to both short-term clinical outcomes, and longer-lasting health effects, systematic characterization of the gut microbiome in pediatric patients during, and beyond, treatment is warranted.

## Introduction

The healthy gastrointestinal tract (GIT) contains a large and complex microbial community of commensal bacteria, archaea, eukaryotes, and viruses, which is referred to as the GIT microbiome. The GIT microbiome benefits the host (Bäckhed et al., 2005; Ley et al., 2006; Ley et al., 2008) through beneficial interactions between the GIT microbiome and the host, These interactions include, facilitating metabolic functions (nutrient production, and digestion), providing protection from pathogens, and participating in immune-system regulation (Bäckhed et al., 2005; Ley et al., 2006; Finke, 2009; Hill and Artis, 2010; Cheng et al., 2019; Khan et al., 2021). The GIT microbiome is now thought to affect most host physiological functions, either directly or indirectly. When the GIT microbiome is disturbed it can result in an imbalance in the normal physiological relationship between host and microbiome, often referred to as dysbiosis. When in a dysbiotic state, healthy host functions can be compromised, either due to a reduction in beneficial bacteria and their metabolic products (*e.g*., butyrate production) or to an increase in opportunistic pathogens. Some combination of these changes is thought to contribute to a variety of diseases (Bäckhed et al., 2004; Manichanh et al., 2012; Morgan et al., 2012; Aydin, 2017; Akshintala et al., 2019; Gérard and Vidal, 2019). Factors that contribute to changes in the microbiome include age, diet, medication, infection, disease, host genetic factors, immune response, and environmental exposure. However, healthy human microbiomes are naturally dynamic in composition and not all these factors will necessarily play a role in the origin of dysbiosis.

The composition of the GIT microbiome impacts both the pathogenic development of cancer, as well as the efficacy of treatments (Alexander et al., 2017; Geller et al., 2017; Chau et al., 2021; Nearing et al., 2019; Thomas et al., 2019; Huang et al., 2020; Lucafò et al., 2020; Rotz and Dandoy, 2020; Song et al., 2020). This relationship is bidirectional in that both the treatments of cancer, and the pathological state can influence the composition of the GIT microbiome (Rajagopala et al., 2016; Helmink et al., 2019; Oldenburg et al., 2021). As such, dysbiosis can both cause, and be a result of, cancer. Therefore, understanding and characterizing the GIT microbiome in pediatric cancers, and how it changes with treatment, has broad implications in clinical management. The typical approach involves measuring bacterial genes present in the stool, which is easier to collect and serves as a proxy for the GIT (for this reason the term GIT microbiome, for both this study and the broader literature, will refer to a microbiome inferred from a stool sample). Assessing the GIT microbiome is especially relevant when microbial activities and immune function are interdependent (Frank et al., 2007; Mukherjee et al., 2014; Khosravi et al., 2014; Shi et al., 2017; Iwamura et al., 2017). Mouse studies have demonstrated that regulation of hematopoiesis depends on sensing of microbes (Khosravi et al., 2014; Iwamura et al., 2017). In hematologic malignancies there can be both dysbiosis of the GIT microbiota and dysregulation of the immune system (see Uribe-Herranz et al., 2021 for review). We believe that improved knowledge of microbiome changes resulting from cancer treatments (*e.g*., prophylactic use of antifungals and antacids), and their impact on the physiological relationship between host and microbiome, could inform clinical decision-making to optimize patient outcomes.

The pediatric population is particularly challenging because the composition of the microbiome is age dependent, with some of the most pronounced changes occurring in the first three years of life (Palmer et al., 2007; Koenig et al., 2011; Yatsunenko et al., 2012). The microbiome becomes more stable after the age of three in which there is a shift towards a more adult-like microbiome (Palmer et al., 2007; Koenig et al., 2011; Yatsunenko et al., 2012). Compositional changes that occur during this time include increasing alpha-diversity, increased *Bacteroides* and species of the Firmicutes phylum, and a decrease in Proteobacteria and bifidobacteria (Palmer et al., 2007; Koenig et al., 2011; Yatsunenko et al., 2012). For this reason, it is important to differentiate between patient populations above and below three-years of age when characterizing the effect of a clinical intervention on the patient microbiome.

Children with hematologic malignancies exhibit GIT microbiome dysbiosis compared to healthy controls (Liu et al., 2020). This difference is, in part, due to exposure to chemotherapeutic agents. As patients undergo chemotherapy the composition of their microbiomes change significantly compared to its state prior to treatment (Rajagopala et al., 2016). Changes attributed to chemotherapeutic agents include loss of diversity, and decreases in Firmicutes taxa *Anaerostipes*, *Coprococcus*, *Roseburia*, and *Ruminococcus* (Rajagopala et al., 2016).

Exposure to anti-infectives including antibiotics and antifungals also contributes to the origin of dysbiosis. Patients with hematologic malignancies have immune dysfunction and are particularly susceptible to infectious disease. As such, pediatric hematologic cancer patients receive prophylactic antibiotic treatment (Lehrnbecher et al., 2020). Patients who develop life-threatening infections, and febrile neutropenia, are often treated with additional broad spectrum empiric anti-infectives including antibiotics and/or antifungals (Rasmy et al., 2016). While broad spectrum anti-infectives are critical for patient survival in the short term, they can further dysregulate their GIT microbiota and thereby increase the probability of life-threatening infections over the longer term (Oldenburg et al., 2021). The interactive effect of chemotherapy and additional broad spectrum anti-infectives on GIT dysbiosis has not been investigated in pediatric hematologic cancer patients.

The progression and outcome of hematologic cancer has been associated with a patient’s stool microbiome composition (Montassier et al., 2016; Hakim et al., 2018; Nearing et al., 2019; Jain et al., 2021; Doocey et al., 2022). For example, certain microbes present in the stool of pediatric hematologic cancer patients prior to induction chemotherapy may serve as biomarkers to predict infectious complications and febrile neutropenia in subsequent phases of treatment (Hakim et al., 2018; Nearing et al., 2019). Furthermore, the diversity and relative abundance of microbes in the stool has been linked to how patients with hematologic cancers respond to chemotherapy, hematopoietic stem cell transplants, and monoclonal antibody therapy (Galloway-Peña et al., 2017). Thus, the potential to optimize the clinical management of hematologic cancers will depend on improved knowledge of both (i) GIT microbial dysbiosis (as inferred from stool) associated with hematologic cancers and (ii) changes to the microbiome that result from the interactive effects of common cancer treatments.

Here we examine the impact of age, antibiotic, and antifungal exposure on the microbiome composition of patients undergoing similar chemotherapeutic treatments. We hypothesize that patients under three will have a more limited microbiome and different composition than those over three. In addition we hypothesize that exposure to antibiotics, and antifungals will result in decreased diversity and compositional changes. We will assess the impact of these variables on the stool microbiome separately and in combination, and examine what role the identified taxonomic changes might play. We will use both 16S rRNA and auxiliary taxonomic markers derived from metagenome sequence data to assess compositional differences between these patient groups and examine concordance between these different markers.

## Methods

We investigated 134 stool samples collected throughout the course of treatment from 47 pediatric patients undergoing care at the IWK Health Centre, Nova Scotia, Canada with leukemia, and lymphoma (**table 1**). Among the 47 patients were 33 acute lymphoblastic leukemia (ALL), 5 acute myeloid leukemia (AML), 4 hodgkin’s lymphoma (HL), and 5 non-hodgkin’s lymphoma (NHL) patients. Ninety-seven stool samples were collected from the 33 ALL patients (1 - 11 samples per patient average 2.9 samples). Twenty-two stool samples from the 5 AML patients (1-11 per patient, mean 4.4), five stool samples from the 4 HL patients (1-2 per patient, mean 1.25), and 10 stool samples from the 5 NHL patients (1-3 samples per patient mean 2). Data on age, sex, antibiotic, and antifungal use, and days from start of chemotherapy were recorded for each sample.

Stool samples were stored at -20°C immediately following collection and for transport, and were transferred to a -80°C freezer until analysis. Total stool DNA was extracted from each sample using QIAGEN PowerFecal DNA kit. Both 16S rRNA amplicon sequencing and whole genome shotgun metagenomic sequencing were performed on the extracted DNA.

### 16S rRNA gene data

The V4-V5 variable region of the 16S rRNA gene was amplified by using polymerase chain reaction from extracted DNA using conditions and primers outlined in the Microbiome Helper protocol (Comeau et. al., 2017). The amplified products were sequenced on the Illumina MiSeq (paired-end 300 bp) at the Integrated Microbiome Resource of Dalhousie University, Nova Scotia, Canada. Analysis of 16S rRNA gene sequences employed the standard Microbiome Helper workflow pipeline (Comeau et al., 2017). Specifically, primers were removed using cutadapt (Martin, 2011) and sequences were imported into QIIME2 (Bolyen et al., 2019). Paired-end reads were joined using VSEARCH (Rognes et. al., 2016), and deblur (Amir et. al., 2017), with a trim length of 360 nucleotides, was used to correct reads and obtain amplicon sequence variants (ASVs). ASVs account for sequencing error by considering frequencies of sequences and result in a single sequence, which can be compared to future samples. ASVs with a frequency less then 0.1% of mean sequence depth were removed. MAFFT (Katoh and Standley, 2013) was used to align ASV sequences and SEPP-tree (Price et al., 2009) for tree construction. Taxonomic assignment was done with classify-sklearn in QIIME2 using the SILVA rRNA database (SILVA-132-99-16S_V4.V5_515F_926R) (Quast et al., 2013). ASVs were collapsed to species levels assignments for subsequent analyses using QIIME2 “taxa collapse” option with level set to 7. Only species that occurred in 10% of samples were used in subsequent analyses.

### Shotgun metagenome data

Shotgun metagenome sequences were obtained using Nextera XT (Illumina) libraries prepared from purified DNA. Libraries were pooled and subjected to paired-end NextSeq (Illumina Hi-Output 300 cycle kit) sequencing (150 bp). Sample processing followed the standard operating procedure outlined in microbiome helper (Comeau et al., 2017). The kneaddata pipeline was used with Trimmomatic (Bolger et al., 2014) to remove low quality sequence reads (reads < 50 base pairs, and with PHRED<Q20 were removed), and human and PhiX174 contaminates were removed using Bowtie2 (Langmead and Salzberg, 2012). MetaPhlAn3 (Beghini et al., 2021) was used to profile the taxonomic composition of the microbial community from the sequence read data, and read counts of taxa for each sample were obtained using the “–t rel_ab_w_reads_stats” option. Only species that occurred in 10% of samples were used in downstream analyses.

The paired trimmed quality controlled and decontaminated sequences were concatenated, and HUMAnN3 (Beghini et al., 2021) was used to assign reads to Metacyc pathways (Caspi et al., 2020). The pathway counts files for each sample were combined and normalized as counts per million sequence reads. Only pathways that occurred in 10% of samples were used in downstream analyses.

### Analyses

Relative abundance of taxa was assessed for both datasets. Alpha-diversity (Shannon diversity, Chao-1, and Faith’s phylogenetic diversity) was obtained from count data for both 16S rDNA (16S) ASVs, and metagenome sequence (MGS) species data using the R packages phyloseq (McMurdie and Holmes, 2013) and vegan (Oksanen et al., 2020). Changes in alpha diversity (Shannon diversity, Chao-1, and Faith’s phylogenetic diversity) with age, antibiotic use, antifungal use, sex, treatment time, and cancer type were assessed (Wilcoxon test or Kruskal-Wallis test) for significance (*α*<0.05).

Differential abundance analyses were performed on count data to examine if specific taxa were associated with metadata variables of interest. As there is no consensus opinion on the method of analysis for this type of data, and as different methodologies can yield differing results, we followed the recent recommendation of Nearing and co authors (2022) and utilized several methods to assess robustness of the taxa identified. Methods examined included: 1.) The Wilcoxon test on CLR transformed data. 2.) An ANOVA-like differential expression (ALDEx2) (Fernandes et al., 2014) analysis using CLR transformation accounting for the geometric mean of the denominator two ways (all features and inter-quartile log ratio). 3.) Linear discriminant analysis with effect size (LEfSe) (Segata et al., 2011). 4.) Microbiome multivariate association with linear models (MaAsLin2) (Mallick et. al., 2021), using either default normalization and transformation (TSS and LOG) or using the CLR transformed data. Note that for methods 1, 2 & 4 the significance threshold was adjusted according to the Benjamini-Hochberg (BH) method (Benjamini and Hochberg, 1995) to control the expected proportion of false discoveries (FDR). For method 3 we started with the *p*-values computed by LEfSe, filtered them according to the BH method, and then restricted our selection criteria to a linear discriminate value >2 among the subset inferred to control the FDR (α<0.05). Note that these methods differ in whether they account for the compositional nature of the data, how transformation and normalization of the data were performed, and what statistical distributions were used, for a detailed comparisons see Nearing et al. (2022). The methods of Wilcoxon (CLR), ALDEx2 (CLR), and MaAsLin2 (CLR) take the ratio of read counts of all taxa within a sample as the reference for that sample to account for the compositional nature of the data, while the other methods do not. All four methods were used to separately analyze age, antibiotic use, and antifungal use. In addition, we utilized MaAsLin2 with the CLR transformation to carry out a joint analysis of the variables simultaneously for association with the microbial community composition and to examine functional changes.

## Results

### Patient population

The 134 samples from 47 patients were grouped by age at diagnosis. Forty-nine stool samples from 11 patients were under the age of 3 (U3) and 85 stool samples from 36 patients were over the age of 3 (O3). Males and females were represented equally, with 49% of patients and 48% of stool samples collected from males. The collection of stool samples occurred at various time points during chemotherapy. The average number of days between start of chemotherapy and stool sample collection was 80.5 and ranged from 5 days before to 471 days after start of chemotherapy (**Table 1**).

**Table 1:** Patient and sample population information.

	Total	ALL	AML	HL	NHL
**Patient information**					
**Number of patients**	47	33	5	4	5
**Male**	23	17	2	2	2
**under 3**	11	8	2	0	1
**antibiotic use**	43	32	5	2	4
**antifungal use**	29	21	5	0	3
**average courses of antibiotic (min;max)**	7.1 (0;28)	7.0 (0;28)	14.4 (5;25)	1.25 (0;3)	5.6 (0;13)
**average courses of antifungal (min;max)**	2.8 (0;16)	2.3 (0;8)	8.6 (5;16)	0	2.6 (0;5)
**patients with pre-treatment samples**	21	13	5	2	1
**patients with first 30 days samples**	30	21	3	2	4
**patients with day 31-180 samples**	20	13	3	1	3
**patients with beyond 180 days samples**	13	12	1	0	0
**Stool information**					
**Stool samples collected**	134	97	22	5	10
**Male samples**	64	52	3	3	6
**average number of samples per patient (min;max)**	2.9 (1;11)	2.9 (1;11)	4.4 (1;11)	1.3 (1;2)	2.0 (1;3)
**under 3 samples**	49	40	8	0	1
**samples with antibiotic use 15 days prior**	71	44	20	2	5
**samples with antifungal use 15 days prior**	46	25	16	0	5
**pre-treatment samples**	22	13	6	2	1
**first 30 days samples**	41	31	3	2	5
**day 31-180 samples**	52	35	12	1	4
**samples beyond 180 days**	19	18	1	0	0
**average number of day between start of chemotherapy and stool sample (min;max)**	80.5 (-5;471)	94.2 (-5;471)	58.5 (-5;183)	14.4 (-5;38)	29.7 (-1; 91)

Prophylactic antibiotic use (septra - sulfamethoxazole/trimethoprim) occurred in all patients and was not included in the count of additional antibiotic use within this study as it was continuously present. Anti-infective status corresponded to antibiotics and antifungals given to patients (see **Table 2**) required for treating infections and or febrile neutropenia episodes as per institutional protocols. Beyond prophylactic antibiotic use, 4 patients received no additional antibiotics while the remaining 43 patients received between 1 and 28 courses of antibiotics with an average of 7.8 courses (average 7.1 when patients with no antibiotic use were included; **Table 1**). Twenty-nine patients received antifungal treatment, while 18 patients did not. From one to 16 courses of antifungals were used among patients treated with an average of 4.5 courses (average 2.8 courses when patients with no antifungal use were included; **Table 1**). The 4 patients that received no additional antibiotics also received no antifungals, while the remaining 14 patients that did not receive antifungals received between 1 and 6 courses of antibiotics with an average of 3.1 courses of antibiotics.

**Table 2:** List of additional antibiotic and antifungals given to patients.

Antibiotics used	Antifungals used
Gentamicin sulfate	Voriconazole
Tobramycin sulfate	Fluconazole
Vancomycin	Caspofungin acetate
Azithromycin	Clotrimazole
Clarithromycin	Amphotericin B Liposomal
Amoxicillin	Pentamidine isethionate
Amoxicillin and clavulanate	
Ampicillin	
Piperacillin and tazobactam sodium	
Cloxacillin	
Meropenem	
Metronidazole	
Cefazolin	
Cefixime	
Cefotaxime	
Ceftazidime	
Ceftriaxone	
Cephalexin	
Ciprofloxacin	
Levofloxacin	
Clindamycin	

“Antibiotic use”, in this study, refers specifically to intervention with additional antibiotics in the 15 days before stool sample collection. Among the 134 stool samples collected, 71 samples were assigned to the antibiotic use group (ab+) and the remaining 63 samples to no antibiotic use (ab-). Likewise, “antifungal use” refers specifically to a prescribed antifungal intervention in the 15 days before stool sample collection. Among the 134 stool samples collected 46 samples were assigned to the antifungal use group (af+) and the remaining 88 samples to no antifungal use (af-). Among the stool samples collected 47 were ab- and af-, 41 were ab+ and af-, 16 were ab- and af+, and 30 were ab+ and af+.

### Inferred taxonomic and functional composition of patient stool microbiomes

Analysis of the 16S rDNA data identified 1184 feature ASVs after 0.1% filtering, in the 134 stool samples. Mean ASV reads per sample was 24,757 (min: 1522; max: 81,140; median: 23,063). Species level assignment (level-7 QIIME2) of ASVs resulted in 489 taxa with 413 occurring in more than one sample and 173 present in 10% of the samples. The average number of species per sample was 61 (min: 6; max: 167; median: 49.5).

Analysis of alternative taxonomic markers derived from the MGS data identified 402 species in the 134 stool samples. Among these species, 325 were found in more than one sample, with 126 present in 10% of samples. The mean number of reads mapped to clades was 3,788,329 with a minimum of 60,159, max of 15,817,214, and median of 2,709,516. Average number of species per sample was 39 (min: 6; max: 99; median: 36).

Analysis and assignment of the metagenome sequence data to Metacyc (Caspi et al., 2020) functional pathways was carried out using HUMAnN3 (Beghini et al., 2021), and identified 506 pathways in the 134 stool samples. Most pathways (482) were found in more than one sample, with 357 pathways present in 10% of samples. The average number of pathways per sample was 238 (min: 94; max 375; median: 232.5).

### Relative abundance and diversity

**Figure 1:**
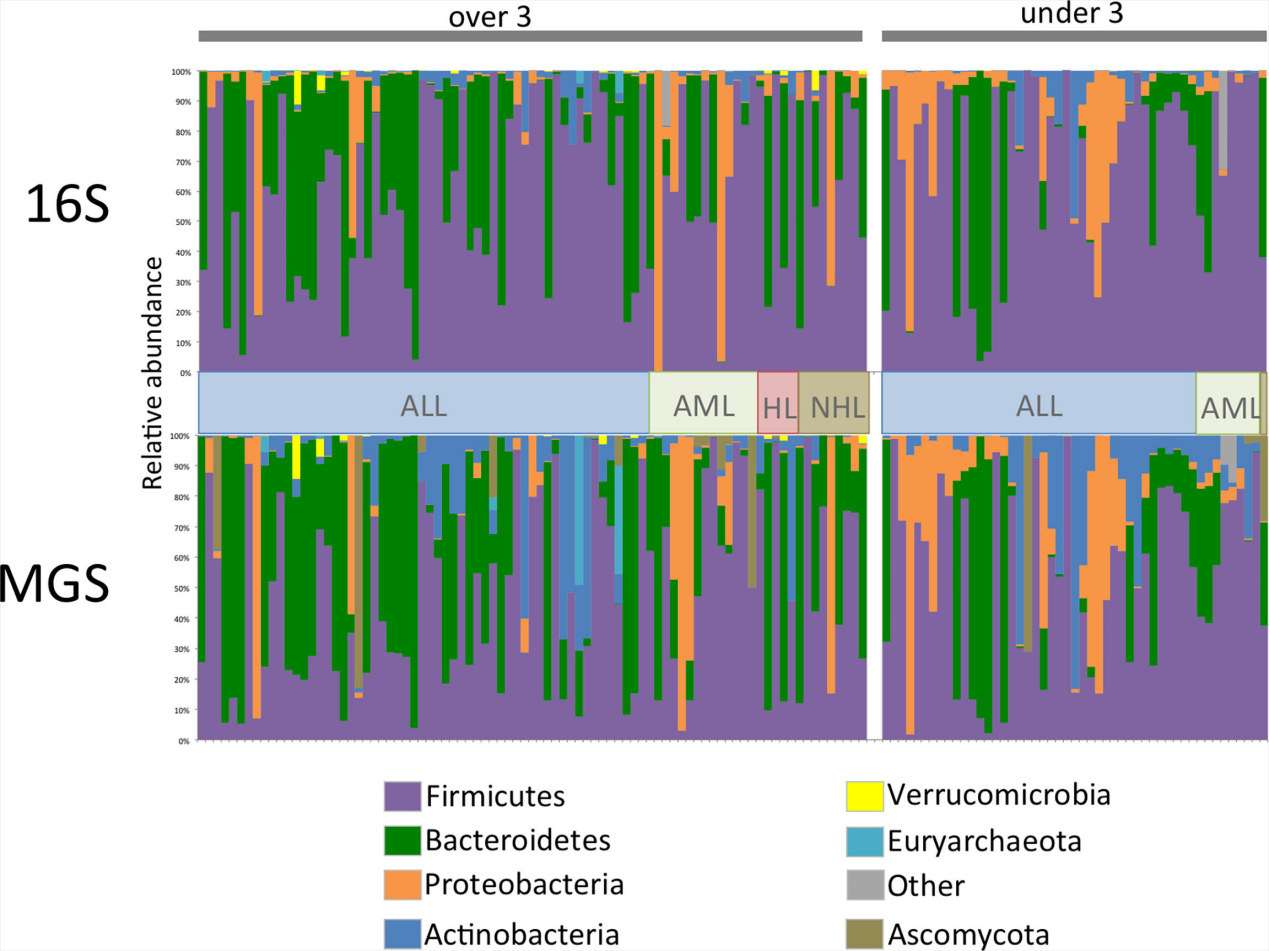
Phylum level relative abundance plot for 134 stool samples collected from 47 patients for 16S rRNA (16S) gene and whole shotgun metagenome sequence (MGS) data. Data is separated by age category and cancer type, ALL, acute lymphoblastic leukemia; AML, acute myeloid leukemia; HL, hodgkin’s lymphoma; NHL, non-hodgkin’s lymphoma. For ease of comparison samples are in the same order in both datasets.

**Figure 2:**
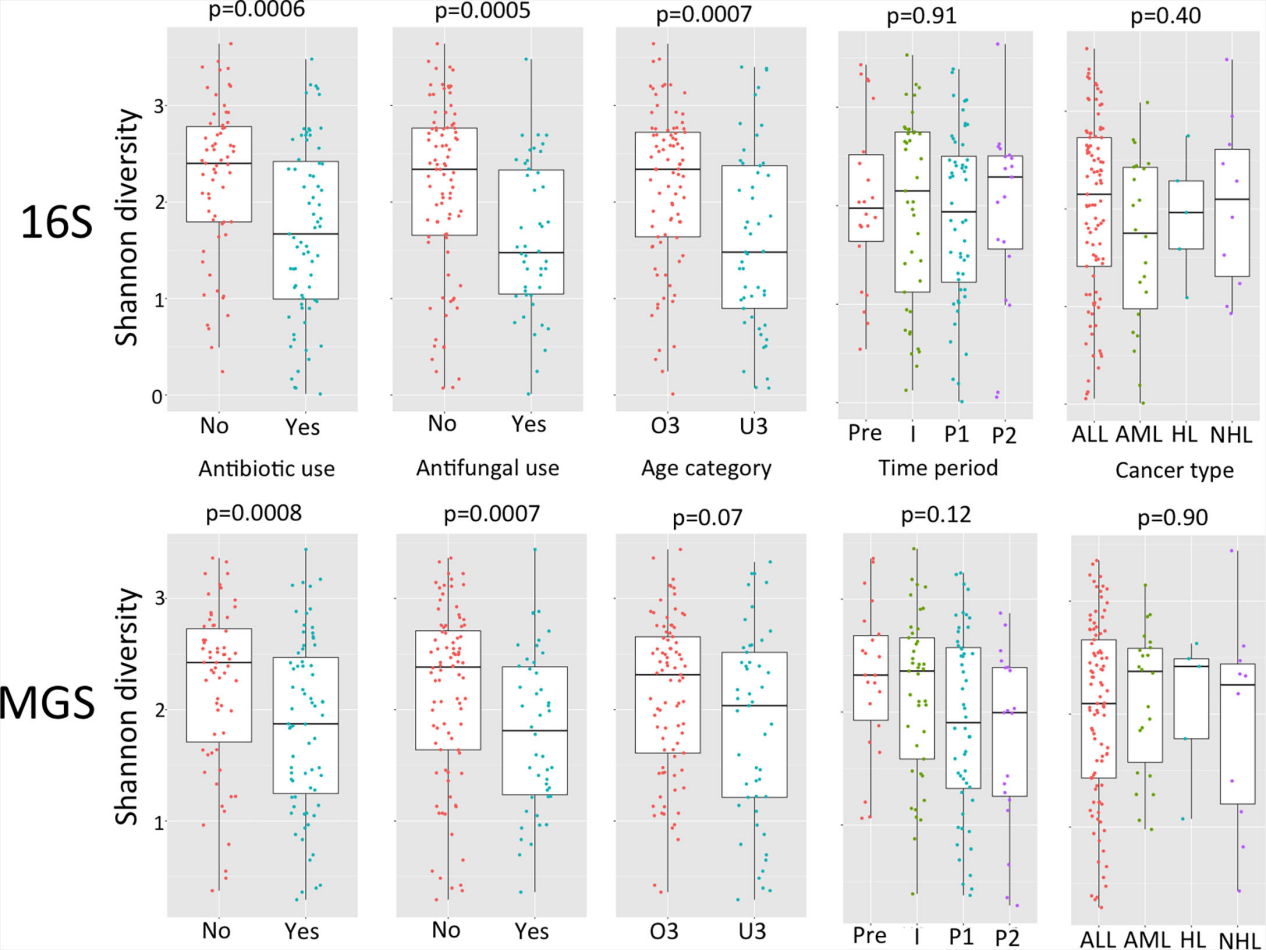
Comparison of alpha diversity using Shannon diversity for 16S rRNA gene (16S) and whole shotgun metagenome data (MGS) for antibiotic use, antifungal use, age category (O3, over 3 years of age; U3, under 3 years of age), time period of treatment (Pre, pre-chemotherapy days -5 to 0; I, induction days 1 to 30; P1, post induction days 31 to 180; P2 post induction days 181+), cancer type (ALL, acute lymphoblastic leukemia; AML, acute myeloid leukemia; HL, hodgkin’s lymphoma; NHL, non-hodgkin’s lymphoma). P values are calculated using Wilcoxon test for pairwise and Kruskall-Wallis for multiple comparisons.

Comparison of relative abundance plots between 16S and MGS data, at the phyla level (**Figure 1**), show more Actinobacteria identified in metagenome sequences than in 16S sequence data whereas the relative frequency of most other phyla were similar. Diversity analyses were performed on rarefied data using the Shannon diversity measure, with either Wilcoxon rank sum or Kruskal Wallis test employed to test for a significant change in diversity between groups of interest. Shannon diversity measures for both 16S and MGS data were significantly decreased with antibiotic (*p*=0.0006; *p*=0.008) and antifungal (*p*=0.0005; *p*=0.007) use (**Figure 2**), while diversity in the under 3 age group was significantly decreased for 16S data (*p*=0.0007) but only marginally decreased for MGS data (*p*=0.07) (**Figure 2**). Sex (*p*=0.68; *p*=0.73), type of cancer (*p*=0.40; *p*=0.90), and treatment period (*p*=0.91; *p*=0.12) did not differ significantly in diversity in either dataset (**Figure 2**). We also analyzed Chao-1 and Faith’s phylogenetic diversity. These showed the same diversity patterns and significance relationships as inferred under Shannon diversity, but with one exception. The difference involved treatment period, which was significant in both datasets (p=0.0053; p=0.031) under the Chao-1 and Faith metrics (**Supplemental Figures 1, 2**).

### Differential abundance analyses

We performed six tests to examine differential abundance (Wilcoxon-CLR, ALDEx2-denom, ALDEx2-iqlr, LEfSe, MaAsLin2-default, and MaAsLin2-CLR). These tests were applied to the count data from 16S and MGS datasets with respect to age category, antibiotic use, and antifungal use. MaAsLin2-CLR was applied to 16S and MGS datasets for a combination of factors.

For taxonomic association with age category (O3 vs U3) the analyses identified from 6 to 54 taxa in the 16S dataset and from 10 to 36 taxa in the MGS dataset, depending on the test method (**Table 3**). For taxonomic association with antibiotic use, these analyses identified from 9 to 42 taxa in the 16S dataset and from 9 to 40 taxa in the MGS dataset (**Table 3**). For taxonomic association with exposure to antifungals, these analyses identified from 11 to 83 taxa in the 16S dataset and from 21 to 59 in the MGS dataset (**Table 3**). These results highlight the impact that the analytical method has on findings and is consistent with Nearing et al. (2022), which found ALDEx2 to consistently identify fewer significant taxa, while LEfSe and Wilcoxon identify the most taxa as significant. Note that the standard method of LEfSe analysis is intended for biomarker discovery, and will typically identify the max number of taxa. However, in this study our LEfSe selection criterion includes a step that controls the FDR; hence, the number of identified taxa is greatly decreased, yielding results consistent with the other methods which apply a FDR correction.

**Table 3:** Number of taxa identified in differential abundance analyses at a BH corrected p value <0.05 for different groups in the 16S rRNA (16S) and metagenome sequence (MGS) dataset.

Analysis	age category	antibiotic use	antifungal use
**Wilcoxon clr**			
** 16S**	54	40	60
** MGS**	36	40	52
**ALDEx2-iqlr**			
** 16S**	6	9	11
** MGS**	10	9	21
**ALDEx2-denom**			
** 16S**	7	10	13
** MGS**	13	14	25
**LEfSe^a,b^**			
** 16S**	43	28	83
** MGS**	17	25	39
**MaAsLin-default**			
** 16S**	49	42	83
** MGS**	19	37	59
**MaAsLin2-clr**			
** 16S**	23	34	37
** MGS**	23	37	42

a LEfSe works at multiple taxonomic levels, only Species level significant counts are shown, additional taxonomic levels were also significant. ^b^p-values computed by LEfSe were filtered according to the BH method, and then we restricted our selection criteria to a linear discriminate value >2 among the subset inferred to control the FDR (α<0.05).

### Age-related taxonomic associations

Examination of difference in the microbiome based on age at diagnosis identified an increased diversity in the O3 relative to U3 samples. This was more evident with the 16S data compared with the MGS data (**Figure 2**). Within the 16S data 99 taxa were identified in one or more test. Among these 99 taxa, 26 were increased in U3 (3 Actinobacteria, 1 Bacteroidetes, 19 Firmicutes, 1 Fusobacteria, and 2 Proteobacteria) and 73 were increased in O3 (2 Actinobacteria, 13 Bacteroidetes, 55 Firmicutes, 2 Proteobacteria, and 1 Verrucomicrobia) (**Supplemental Table 1**). In the MGS dataset 34 taxa were identified in one or more test as differing between age groups, with 20 increased in U3 (4 Actinobacteria, 14 Firmicutes, and 2 Proteobacteria) and 14 increased in O3 (1 Actinobacteria, 8 Bacteroidetes, and 5 Firmicutes) (**Supplemental Table 2**).

Using the criterion of concordance among 4 or more significance tests, we identified 11 taxa with increased relative abundance in O3 (16S = 5 taxa; MGS = 6 taxa; **Table 4**). Among these eleven, members of three taxonomic lineages were shared between datasets: (i) *Alistipes* (*A.sp, A. putredinis*) in the phylum Bacteroidetes; (ii) *Parabacteroides* (*P. sp, P. distasonis*) in the phylum Bacteroidetes; and (iii) *Ruminococcus* (*R. sp, R. bromii*) in the phylum Firmicutes. Note that analysis of the intersection between 16S and MGS is constrained by their different levels of taxonomic resolution. This is why, for each above lineage, one was resolved to a named species (MGS) and one was unresolved (16S; indicated by “sp”). The 16S data identified 2 additional Firmicutes as increased in O3 (*C. spiroforme, and Ruminococcaceae Incertae Sedis sp*). The MGS data also identified additional taxa increased in O3 (**Table 4**); 2 additional *Bacteroides* (*B. ovatus, B. uniformis*), and an Actinobacteria, *Collinsella aerofaciens*.

**Table 4:** Taxa identified as significantly increased in 4 or more differential abundance analysis methods based on age category (over 3 years O3 and under 3 years U3 of age) using 16S and MGS data.

Phylum	Class	Family	taxa	W	Ad	Ai	L	Md	Mc	data
**O3**										
**Actinobacteria**	Coriobacteriia	Coriobacteriaceae	*Collinsella aerofaciens*	x	x		x	x	x	MGS
**Bacteroidetes**	Bacteroidia	Rikenellaceae	*Alistipes putredinis*	x	x		x	x	x	MGS
**Bacteroidetes**	Bacteroidia	Rikenellaceae	*Alistipes* sp.	x	x	x	x	x	x	16S
**Bacteroidetes**	Bacteroidia	Bacteroidaceae	*Bacteroides ovatus*	x	x	x	x	x	x	MGS
**Bacteroidetes**	Bacteroidia	Bacteroidaceae	*Bacteroides uniformis*	x	x	x	x	x	x	MGS
**Bacteroidetes**	Bacteroidia	Tannerellaceae	*Parabacteroides distasonis*	x	x		x	x	x	MGS
**Bacteroidetes**	Bacteroidia	Tannerellaceae	*Parabacteroides* sp.	x			x	x	x	16S
**Firmicutes**	Bacilli	Erysipelatoclostridiaceae	*Clostridium spiroforme*	x	x		x	x	x	16S
**Firmicutes**	Clostridia	Ruminococcaceae	*Ruminococcaceae Incertae Sedis* sp.	x			x	x	x	16S
**Firmicutes**	Clostridia	Ruminococcaceae	*Ruminococcus* sp.	x			x	x	x	16S
**Firmicutes**	Clostridia	Ruminococcaceae	*Ruminococcus bromii*	x	x	x	x	x	x	MGS
**U3**										
**Actinobacteria**	Actinobacteria	Bifidobacteriaceae	*Bifidobacterium breve*	x			x	x	x	MGS
**Firmicutes**	Erysipelotrichia	Erysipelotrichaceae	*Erysipelatoclostridium ramosum*	x	x	x	x	x	x	MGS
**Firmicutes**	Bacilli	Erysipelatoclostridiaceae	*Erysipelatoclostridium* sp.	x	x	x			X	16S
**Firmicutes**	Clostridia	Lachnospiraceae	*Ruminococcus gnavus*	x	x	x	x	x	x	MGS
**Firmicutes**	Clostridia	Lachnospiraceae	*Ruminococcus gnavus group* sp.	x	x	x			x	16S
**Firmicutes**	Bacilli	Streptococcaceae	*Streptococcus* sp.	x	x	x			x	16S
**Firmicutes**	Bacilli	Streptococcaceae	*Streptococcus thermophilus*	x	x	x			x	MGS
**Firmicutes**	Negativicutes	Veillonellaceae	*Veillonella dispar*	x	x	x		x	x	MGS
**Firmicutes**	Negativicutes	Veillonellaceae	*Veillonella parvula*	x	x	x	x	x	x	MGS
**Firmicutes**	Negativicutes	Veillonellaceae	*Veillonella* sp.	x	x	x	x	x	x	16S
**Proteobacteria**	Gammaproteobacteria	Enterobacteriaceae	*Enterobacter cloacae complex*	x	x	x	x		x	MGS
**Proteobacteria**	Gammaproteobacteria	Enterobacteriaceae	*Escherichia coli*	x	x	x	x	x	x	MGS
**Proteobacteria**	Gammaproteobacteria	Enterobacteriaceae	*Escherichia Shigella* sp.	x	x	x	x	x	x	16S

W, Wilcoxon test on CLR transformed data corrected for multiple tests using BH. Ad, ALDEx2 using CLR transformed data with all features used as the denominator for the geometric mean calculations. Ai, ALDEx2 using CLR transformed data using the “iqlr” (features with variance between lower and upper quartile variance) set of features as the denominator for the geometric mean calculation. L, LEfSe analysis using the p value (p< 0.008 16S; p<0.003 MGS) that controls the FDR and an LDA >2, this analysis included all taxonomic levels but only Species level results are shown. Md, MaAsLin2 with default parameters TSS normalization and LOG transformation. Mc, MaAsLin2 analysis on CLR transformed data.

We identified 13 taxa having increased relative abundance in U3 (16S = 5 taxa; MGS = 8 taxa; **Table 4**) according to the criterion of concordance among 4 or more significance tests. Here, members of 5 taxonomic lineages were shared between datasets: (i) *Erysipelatoclostridium* (*E. sp, E. ramosum*); (ii) *Ruminococcus gnavus*; (iii) *Streptococcus* (*S. sp, S. thermophilus*); (iv) *Veillonella* (*V. sp, V. parvula, V. dispar*); and (iv) *Escherichia* (*E. sp*, *E. coli*) (**Table 4**). In only one case was the lineage resolved as the same species, *Ruminococcus gnavus*, by both datasets. In addition MGS identified the Actinobacteria *Bifidobacterium breve*, and a Proteobacteria from the *Enterobacter cloacae complex* as increased in U3.

### Antibiotic related taxonomic associations

Microbiome diversity was significantly lower in the antibiotic use group (ab+) for both types of data (Shannon diversity; **Figure 2**). Within the 16S dataset we identified 73 taxa as having significantly different relative abundance between groups (ab+ vs. ab-) according to one or more of the methods; 40 were lower in the ab+ group (1 Actinobacteria, 38 Firmicutes, and 1 Proteobacteria) and another 33 were higher in that group (4 Actinobacteria, 1 Bacteroidetes, 25 Firmicutes, 1 Fusobacteria, and 2 Proteobacteria) (**Supplemental Table 1**). In the MGS dataset 60 taxa were identified as having significantly different relative abundance between groups in one or more analysis; 28 were lower in the ab+ group (including 5 Actinobacteria, and 23 Firmicutes) and another 32 were higher in the ab+ group (including 5 Actinobacteria, 3 Bacteroidetes, 20 Firmicutes, 3 Proteobacteria, and 1 Ascomycota) (**Supplemental Table 2**).

Using the criterion of concordance among 4 or more significance tests, we identified 17 taxa with lower relative abundance in the ab+ group (16S = 9 taxa; MGS = 8 taxa; **Table 5**). Among these, members of six taxonomic lineages were identified in both datasets: (i) *Anaerostipes* (*A. sp, A. hadrus*), (ii) *Blautia* (*B. sp, B obeum, B.faecis*), (iii) *Dorea* (*D. sp, D. longicatena*), (iv) *Fusicatenibacter* (*F. sp, F. saccharivorans*), (v) *Roseburia* (*R. sp, R. intestinalis*), and (vi)*Ruminococcus gnavus* (**Table 5**). In addition to the above taxa, *Romboutsia sp* and *Subdoligranulum sp* were identified in the 16S dataset and *Agathobaculum butyriciproducens*, and *Coprococcus comes* in the MGS dataset (**Table 5**).

**Table 5:** Taxa identified by 4 or more different methods as being differentially abundant based on antibiotic use in the 15 days before sampling identified in 16S and MGS data.

Phylum	Class	Family	Species	W	Ad	Ai	L	Md	Mc	data
**decreased**										
**Firmicutes**	Clostridia	Lachnospiraceae	*Anaerostipes hadrus*	X	X	X	X	X	X	MGS
**Firmicutes**	Clostridia	Lachnospiraceae	*Anaerostipes* sp.	X	X	X	X	X	X	16S
**Firmicutes**	Clostridia	Lachnospiraceae	*Blautia faecis*	X	X	X	X	X	X	16S
**Firmicutes**	Clostridia	Lachnospiraceae	*Blautia obeum*	X	X	X	X	X	X	MGS
**Firmicutes**	Clostridia	Lachnospiraceae	*Blautia* sp.	X	X	X	X	X	X	16S
**Firmicutes**	Clostridia	Lachnospiraceae	*Coprococcus comes*	X	X	X	X	X	X	MGS
**Firmicutes**	Clostridia	Lachnospiraceae	*Dorea longicatena*	X	X	X	X	X	X	MGS
**Firmicutes**	Clostridia	Lachnospiraceae	*Dorea* sp.		X	X	X	X	X	16S
**Firmicutes**	Clostridia	Lachnospiraceae	*Fusicatenibacter saccharivorans*	X	X	X	X	X	X	MGS
**Firmicutes**	Clostridia	Lachnospiraceae	*Fusicatenibacter* sp.	X	X	X	X	X	X	16S
**Firmicutes**	Clostridia	Lachnospiraceae	*Roseburia intestinalis*	X	X	X	X	X	X	MGS
**Firmicutes**	Clostridia	Lachnospiraceae	*Roseburia* sp.	X	X	X	X	X	X	16S
**Firmicutes**	Clostridia	Lachnospiraceae	*Ruminococcus gnavus*	X	X	X	X	X	X	MGS
**Firmicutes**	Clostridia	Lachnospiraceae	*Ruminococcus gnavus group* sp.	X			X	X	X	16S
**Firmicutes**	Clostridia	Peptostreptococcaceae	*Romboutsia* sp.	X	X	X	X	X	X	16S
**Firmicutes**	Clostridia	Ruminococcaceae	*Agathobaculum butyriciproducens*	X	X	X	X	X	X	MGS
**Firmicutes**	Clostridia	Ruminococcaceae	*Subdoligranulum* sp.	X	X	X	X	X	X	16S
**increased**										
**Actinobacteria**	Actinobacteria	Actinomycetaceae	*Actinomyces sp oral taxon 181*	X	X		X	X	X	MGS
**Actinobacteria**	Actinobacteria	Micrococcaceae	*Rothia mucilaginosa*	X	X		X	X	X	MGS
**Firmicutes**	Bacilli	Aerococcaceae	*Abiotrophia sp HMSC24B09*	X	X		X	X	X	MGS
**Firmicutes**	Bacilli	Aerococcaceae	*Abiotrophia uncultured* sp.	X	X		X	X	X	16S
**Firmicutes**	Bacilli	Enterococcaceae	*Enterococcus* sp.	X	X	X		X	X	16S
**Firmicutes**	Bacilli	Lactobacillaceae	*Lactobacillus rhamnosus*	X	X	X	X	X	X	MGS
**Firmicutes**	Clostridia	Lachnospiraceae	*Clostridium bolteae*	X	X			X	X	MGS

W, Wilcoxon test on CLR transformed data corrected for multiple tests using BH. Ad, ALDEx2 using CLR transformed data with all features used as the denominator for the geometric mean calculations. Ai, ALDEx2 using CLR transformed data using the “iqlr” (features with variance between lower and upper quartile variance) set of features as the denominator for the geometric mean calculation. L, LEfSe analysis using the p value (p <0.004 16S; p<0.008 MGS) that controls the FDR and an LDA >2, this analysis included all taxonomic levels but only Species level results are shown. Md, MaAsLin2 with default parameters TSS normalization and LOG transformation. Mc, MaAsLin2 analysis on CLR transformed data.

We identified a set of seven taxa having higher relative abundance in the ab+ group (16S = 2 taxa; MGS = 5 taxa; **Table 5**) according to the criterion of concordance among 4 or more significance tests. Only *Abiotrophia* (*A. sp, A. uncultured*) was found to have significantly higher relative abundance in both datasets (**Table 5**). The taxon uniquely inferred from the 16S dataset as having higher relative abundance in the ab+ group was *Enterococcus* sp. The four taxa uniquely identified in the MGS data as having higher relative abundance in this group were: (i) *Actinomyces sp oral taxon 181*, (ii) *Rothia mucilaginosa*, (iii) *Clostridium bolteae*, and (iv) *Lactobacillus rhamnosus* (**Table 5**).

### Antifungal-related taxonomic associations

Microbiome diversity was also significantly lower in the group having received antifungals (again, up to 15 days before stool collection) for both types of data (Shannon diversity; **Figure 2**). Within the 16S dataset we identified 134 taxa as having significantly different relative abundance between groups (af+ vs. af-) according to one or more of the methods; 103 taxa were lower (8 Actinobacteria, 15 Bacteroidetes, 76 Firmicutes, 3 Proteobacteria, and 1 Verrumicrobiota) and 31 were higher (2 Actinobacteria, 21 Firmicutes, 1 Fusobacteria, and 7 Proteobacteria) in the af+ group (**Supplemental Table 1**). In the MGS dataset 89 taxa were identified as having significantly different relative abundance between groups according to one or more of the methods; 55 were lower (13 Actinobacteria, 8 Bacteroidetes, 33 Firmicutes, and 1 Ascomycota) and 34 were higher (4 Actinobacteria, 2 Bacteroidetes, 21 Firmicutes, and 7 Proteobacteria) in the af+ group (**Supplemental Table 2**).

Using the criterion of concordance among 4 or more significance tests, we identified 19 taxa with lower relative abundance in the af+ group (16S = 10 taxa; MGS = 9 taxa; **Table 6**). Among these, members of five taxonomic lineages were identified in both datasets: (i) *Alistipes* (*A. sp, A. finegoldii, A. putredinis*), (ii) *Parabacteroides merdae*, (iii) *Anaerostipes* (*A. sp, A. hadrus*), (iv) *Faecalibacterium* (*F. sp, F. prausnitzii*), and (v) *Blautia* (*B. faecis, B. obeum*). The 16S dataset also identified a *Bacteriodes* sp., *Ruminococcus* sp., two species of Oscillospiraceae, and *Subdoligranulum sp* as lower in relative abundance (**Table 6**). The MGS dataset also identified *Bifidobacterium longum*, *Collinsella aerofaciens*, and *Fusicatenibacter saccharivorans*.

**Table 6:** Taxa identified by 4 or more different methods as being differentially abundant based on antifungal use in the 15 days before sampling identified in 16S rDNA and shotgun metagenome sequence data.

Phylum	Class	Family	Species	W	Ad	Ai	L	Md	Mc	data
**decreased**										
**Actinobacteria**	Actinobacteria	Bifidobacteriaceae	*Bifidobacterium longum*	X	X	X	X	X	X	MGS
**Actinobacteria**	Coriobacteriia	Coriobacteriaceae	*Collinsella aerofaciens*	X	X	X	X	X	X	MGS
**Bacteroidetes**	Bacteroidia	Bacteroidaceae	*Bacteroides* sp.	X	X	X	X	X	X	16S
**Bacteroidetes**	Bacteroidia	Rikenellaceae	*Alistipes finegoldii*	X	X	X	X	X	X	MGS
**Bacteroidetes**	Bacteroidia	Rikenellaceae	*Alistipes putredinis*	X	X	X	X	X	X	MGS
**Bacteroidetes**	Bacteroidia	Rikenellaceae	*Alistipes* sp.	X	X	X	X	X	X	16S
**Bacteroidetes**	Bacteroidia	Tannerellaceae	*Parabacteroides merdae*	X	X	X	X	X	X	16S
**Bacteroidetes**	Bacteroidia	Tannerellaceae	*Parabacteroides merdae*	X	X	X	X	X	X	MGS
**Firmicutes**	Clostridia	Lachnospiraceae	*Anaerostipes hadrus*	X	X	X	X	X	X	MGS
**Firmicutes**	Clostridia	Lachnospiraceae	*Anaerostipes* sp.	X	X	X	X	X	X	16S
**Firmicutes**	Clostridia	Lachnospiraceae	*Blautia faecis*	X			X	X	X	16S
**Firmicutes**	Clostridia	Lachnospiraceae	*Blautia obeum*	X	X	X	X	X	X	MGS
**Firmicutes**	Clostridia	Lachnospiraceae	*Fusicatenibacter saccharivorans*	X			X	X	X	MGS
**Firmicutes**	Clostridia	Ruminococcaceae	*Faecalibacterium prausnitzii*	X	X	X	X	X	X	MGS
**Firmicutes**	Clostridia	Ruminococcaceae	*Faecalibacterium* sp.	X	X	X	X	X	X	16S
**Firmicutes**	Clostridia	Ruminococcaceae	*Ruminococcus* sp.	X	X	X	X	X	X	16S
**Firmicutes**	Clostridia	Ruminococcaceae	*Subdoligranulum* sp.	X	X		X	X	X	16S
**Firmicutes**	Clostridia	Oscillospiraceae	*UCG-002* sp.		X		X	X	X	16S
**Firmicutes**	Clostridia	Oscillospiraceae	*Oscillospiraceae* sp.	X	X		X	X	X	16S
**increased**										
**Actinobacteria**	Actinobacteria	Actinomycetaceae	*Actinomyces sp oral taxon 181*	X	X	X			X	MGS
**Actinobacteria**	Actinobacteria	Micrococcaceae	*Rothia mucilaginosa*	X	X	X	X	X	X	MGS
**Actinobacteria**	Actinobacteria	Micrococcaceae	*Rothia sp*	X			X	X	X	16S
**Firmicutes**	Bacilli	Aerococcaceae	*Abiotrophia sp HMSC24B09*	X	X	X		X	X	MGS
**Firmicutes**	Bacilli	Enterococcaceae	*Enterococcus faecalis*	X	X	X	X	X	X	MGS
**Firmicutes**	Bacilli	Enterococcaceae	*Enterococcus faecium*	X	X	X	X	X	X	MGS
**Firmicutes**	Bacilli	Enterococcaceae	*Enterococcus* sp.	X	X	X	X	X	X	16S
**Firmicutes**	Bacilli	Streptococcaceae	*Streptococcus infantis*	X	X	X		X	X	MGS
**Firmicutes**	Bacilli	Streptococcaceae	*Streptococcus peroris*	X	X	X	X	X	X	MGS
**Firmicutes**	Clostridia	Clostridiaceae	*Clostridium paraputrificum*	X	X	X	X	X	X	MGS
**Firmicutes**	Clostridia	Clostridiaceae	*Clostridium paraputrificum*	X	X	X	X	X	X	16S
**Firmicutes**	Clostridia	Peptostreptococcaceae	*Clostridioides difficile*	X	X	X			X	MGS
**Firmicutes**	Clostridia	Peptostreptococcaceae	*Clostridioides* sp.	X	X	X	X		X	16S
**Firmicutes**	Erysipelotrichia	Erysipelotrichaceae	*Erysipelatoclostridium ramosum*		X	X		X	X	MGS
**Firmicutes**	Negativicutes	Veillonellaceae	*Veillonella dispar*	X	X	X			X	MGS
**Firmicutes**	Negativicutes	Veillonellaceae	*Veillonella parvula*	X	X			X	X	MGS
**Proteobacteria**	Gammaproteobacteria	Enterobacteriaceae	*Enterobacter cloacae complex*	X	X			X	X	MGS
**Proteobacteria**	Gammaproteobacteria	Enterobacteriaceae	*Escherichia coli*	X	X	X		X	X	MGS
**Proteobacteria**	Gammaproteobacteria	Enterobacteriaceae	*Klebsiella variicola*	X	X			X	X	MGS
**Proteobacteria**	Gammaproteobacteria	Enterobacteriaceae	*Enterobacteriaceae* sp.	X	X	X		X	X	16S
**Proteobacteria**	Gammaproteobacteria	Pasteurellaceae	*Haemophilus parainfluenzae*	X	X	X		X	X	MGS

W, Wilcoxon test on CLR transformed data corrected for multiple tests using BH. Ad, ALDEx2 using CLR transformed data with all features used as the denominator for the geometric mean calculations. Ai, ALDEx2 using CLR transformed data using the “iqlr” (features with variance between lower and upper quartile variance) set of features as the denominator for the geometric mean calculation. L, LEfSe analysis using the p value (p<0.015 16S; p<0.005 MGS) that controls the FDR and an LDA >2, this analysis included all taxonomic levels but only Species level results are shown. Md, MaasLin2 with default parameters TSS normalization and LOG transformation. Mc, MaasLin2 analysis on CLR transformed data.

We identified a set of 21 taxa having higher relative abundance in the af+ group (16S = 5 taxa; MGS = 16 taxa; **Table 6**) according to the criterion of concordance among 4 or more significance tests. Among these, members of four taxonomic lineages were identified in both datasets: (i) *Rothia* (*R.sp, R. mucilaginosa*), (ii) *Enterococcus* (*E.sp, E. faecalis, E. faecium*), (iii) *Clostridium paraputrificum*, and (iv) *Clostridioides* (formerly *Clostridium C.difficile*, *C.sp*) (**Table 6**). The 16S dataset also identified a species of Enterobacteriaceae as having higher relative abundance in the group treated with antifungals. The MGS dataset identified eleven additional taxa having higher relative abundance in this group (**Table 6**).

### Multivariate analysis of age, antibiotic use and antifungal use

Because age and treatment with either antibiotics or antifungals could have interactive effects on microbiome composition, it can be challenging to jointly interpret the separate analyses described above. Furthermore, applying feature-specific methods to inference involving multiple covariates can have unpredictable effects on inference, although the typical result is excess false positives (Mallick et al., 2021). In this study to guard against spurious discoveries of specific taxa, we used FDR control in our analyses in combination with a strict concordance criterion (>4 methods). However, the cost of this approach is reduced power in the univariate cases, and it does not necessarily control false discoveries when trying to infer taxa that are significantly associated with several variables. MaAsLin2 is designed to maintain statistical power in settings involving multiple covariates while controlling the FDR. Hence, we used multivariate MaAsLin2 to analyze the combined effects of age, antibiotic use and antifungal use.

**Figure 3:**
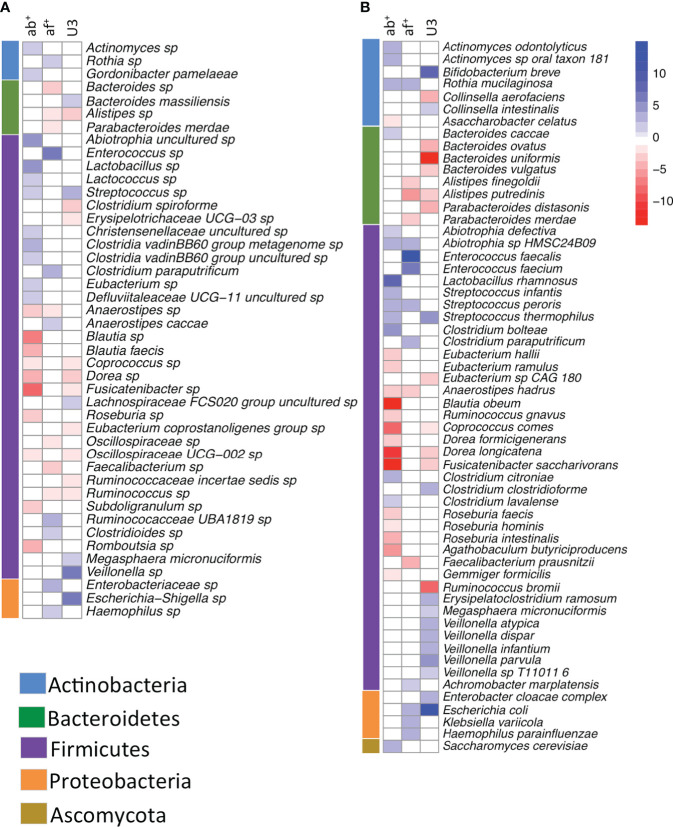
Heatmap showing taxa significantly (FDR corrected p<0.05) associated with antibiotic use, antifungal use and age category in a multivariate analysis using **(A)** 16S rRNA gene data and **(B)** whole shotgun metagenome data. Intensity of change is determined as the negative log of the q-value *coefficient of change, blue indicates taxa increased with antibiotic use (ab+), antifungal use (af+) or in the under 3 age group (U3) and red are taxa decreased with antibiotic use (ab+), antifungal use (af+) or in the under 3 age group (U3). Taxa are sorted by phyla, which are indicated in the bar adjacent to the heatmap.

MaAsLin2 analysis of the 16S data identified 44 taxa (**Figure 3A**) and MGS data identified 58 taxa (**Figure 3B**) as significantly associated in one or more variable. Most taxa identified (36 in the 16S and 48 in the MGS) were significant for a single variable (**Figure 3A, B** 1 shaded box) with antibiotic use associated with the most taxa (16S: 21, MGS: 29), followed by age (16S: 16, MGS: 24), and antifungal use (16S: 15, MGS: 15). We identified an association with multiple variables in eight taxa in 16S and 10 taxa in MGS (**Figure 3A, B** 2 shaded boxes).

For age or antibiotic use, as expected, there were fewer taxonomic associations (**Table 4**, **5**) under the univariate analyses, as they required strict concordance among 4 methods, compared to multivariate MaAsLin2. For age, most of the concordant univariate discoveries described above were recovered within the larger set of taxa identified by 16S MaAsLin2 analysis (7/10 concordant univariate taxa within MaAsLin2 age-associated [n=16]) and MGS MaAsLin2 analysis (13/14 concordant univariate taxa within MaAsLin2 age-associated [n=24]). The same general relationship between the univariate discoveries and the multivariate MaAsLin2 results was observed for antibiotic use (16S: 9/11 within MaAsLin2 [n=21] and MGS: 13/13 within MaAsLin2 [n=29]). However, for antifungal use the number of univariate discoveries were the same (16S: n=15 vs. MaAsLin2: n=15) or more than the number of multivariate MaAsLin2 discoveries (MGS: n=25 vs. MaAsLin2: n=15). This suggests that discovery of taxa associated with more than one variable is likely to be more complicated than predicted simply according to the univariate results. For this reason, identification and interpretation of taxa associated with more than one variable will be inferred only from the multivariate MaAsLin2 results.

Among the lineages associated with more than one variable (n=18), many (n=14) were Firmicutes involved in short chain fatty acid (SCFA) production or lactic acid production (**Figures 3A, B**). Each of the following SCFA producing Firmicutes (*Coprococcus* sp., *C. comes*, *Dorea* sp. *D. longicatena*, *Fusicatinbacter* sp.*, F. saccharivorans*, and *Oscillospiraceae UCG-002*) had significantly lower relative abundance in both U3 and ab+ groups. A SCFA producing Firmicutes, *Ruminococcus* sp., had significantly lower relative abundance in both the U3 and af+ groups. Finally, SCFA-producing *Anaerstipes* (*A. sp*, and *A. hadrus*) had significantly lower relative abundance in both ab+ and af+ groups. The lactic acid producing Firmicutes *Streptococcus sp* and *S. thermophiles* had significantly higher relative abundance in U3 and ab+ groups, and the lactic acid producing Firmicutes *Streptococcus peroris* and *Abiotrophia sp* had significantly higher relative abundance in ab+ and af+ groups. Interestingly, additional SCFA producing Firmicutes had significantly lower relative abundance in either the ab+ or af+ groups and were not associated with age (*e.g*. *Roseburia, Faecalibacterium, Subdoligranulum*) and additional lactic acid producing Firmicutes had significantly higher relative abundance in either ab+ or af+ groups and were not associated with age (*e.g*., *Enterococcus, Lactobacillus*).

Two other patterns are detectable within the MaAsLin2 results (**Figure 3A, B**), that are corroborated by the univariate results. First, several taxa that are significantly more frequent in the ab+ or af+ group are commonly associated with the human oral microbiome (e.g., *Actinomyces, Abiotrophia, Rothia, Streptococcus, Lactococcus*). Furthermore, these lineages represent multiple phyla. Second, several taxa that are significantly more frequent in af+ group are opportunistic pathogens (e.g., *Enterococcus, Clostridium paraputrificum, Klebsiella, Haemophilus*), and represent several phyla.

### Functional multivariate analysis of age, antibiotic use and antifungal use

**Figure 4:**
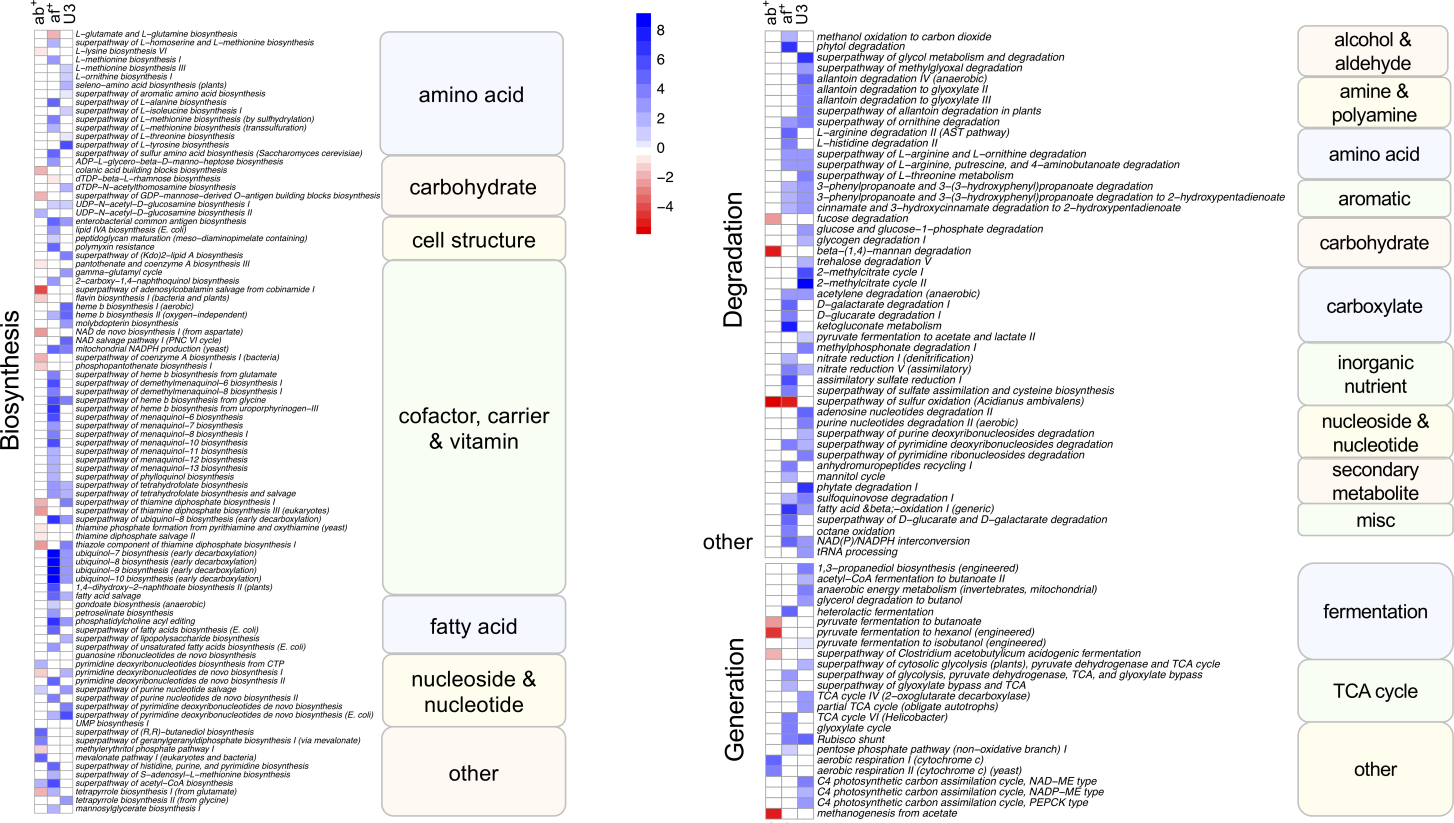
Heatmap showing Metacyc pathways significantly (FDR corrected p<0.05) associated with antibiotic use, antifungal use and age category in a multivariate analysis. Intensity of change is determined as the negative log of the q-value *coefficient of change, blue indicates pathways increased with antibiotic use (ab+), antifungal use (af+) or in the under 3 age group (U3) and red are pathways decreased with antibiotic use (ab+), antifungal use (af+) or in the under 3 age group (U3). Pathways are sorted into Metacyc superclasses, and classes. Biosynthesis refers to biosynthesis pathyways; degradation refers to degradation utilization and assimilation pathways, and generation refers to generation of precursor metabolites and energy pathways.

To assess if changes in the taxonomic composition resulted in changes in functional pathways with age, antibiotic use and antifungal use we performed multivariate analysis using MaAsLin2 using functional pathway data. We identified 165 pathways as significantly associated with one or more variable. Like the taxonomic analysis most pathways (130) were associated with just one variable (**Figure 4**, single shaded box), however it differed from the taxonomic analysis in that antifungal use was associated with the most pathways (57) followed by age (47) and antibiotic use had the least associations (26). An association with multiple variables was identified in 35 pathways (**Figure 4**, 2 shaded boxes), with most of these (28) shared between antifungal use and age, while just 4 pathways were associated with both age and antibiotic use and 3 with both antifungal and antibiotic use. Pathways were predominately of one of three types: biosynthesis pathways (92), degradation/utilization/assimilation pathways, (hereafter, collectively referred to as degradation pathways: 47), and generation of precursor metabolites and energy pathways, (hereafter, collectively referred to as generation pathways: 24).

The largest number of significant pathways (88) was associated with antifungal use, with 83 pathways increased in relative abundance in those samples (**Figure 4**). The 83 increased pathways were predominately involved in biosynthesis pathways (50) followed by degradation pathways (25) and generation pathways (7), while the 5 decreased pathways were predominately biosynthesis pathways (4), with the remaining pathway involved in degradation. We identified 79 pathways that were increased in the U3 samples (35 biosynthesis; 30 degradation; 12 generation; and 2 other) (**Figure 4**). We identified 33 pathways associated with antibiotic use, with 24 of these significantly decreased in relative abundance with antibiotic use compared to no antibiotic use. Most of these (17) were biosynthesis pathways, but also included generation (4) and degradation pathways (3). Among the 9 pathways increased in relative abundance with antibiotic use 7 were biosynthesis pathways and the remaining 2 were generation pathways.

## Discussion

It is well-known that the GIT microbial community is sensitive to many intrinsic and extrinsic factors, including age, antibiotic, and chemotherapy treatment (Palmer et al., 2007; Mangin et al., 2010; Dethlefsen and Relman, 2011; Koenig et al., 2011; Fouhy et al., 2012; Yatsunenko et al., 2012; Bokulich et al., 2016; Korpela et al., 2016; Rajagopala et al., 2016; Oldenburg et al., 2021). Here, we extend this important line of inquiry by investigating the relationship between microbiome composition and three clinically important variables (age, antibiotic exposure, and antifungal exposure) in a pediatric hematological cancer patient population consisting of leukemia and lymphoma patients from Maritime Canada. We found significant differences in microbial diversity due to age, antibiotic use, and antifungal use, and no difference between types of cancers or period of treatment.

We utilized both a portion of the 16S (V4V5) and MGS data to examine the taxa present in stool samples, which we treated as a proxy for what was occurring in the GIT. As each source of taxonomic information has its pros and cons, we chose to examine their concordance. We found general agreement between these two datasets. Notwithstanding that 16S data doesn’t typically identify taxa to species level, whereas MGS does, we found general agreement between the two types of data for taxonomic identification down to the genus level. There were some disagreements between the two datasets, which may be due to the choice of variable region selected, amplification bias, and or taxa present in the reference databases used for each. One of these was the increased relative abundance of Actinobacteria in the MGS data, as compared to the 16S data. Examination of the entire 16S rRNA gene in the future, which is now cost effective, may be useful to reassess these differences along with others and may allow for more detailed resolution of taxa for comparison.

The microbiome and its diversity changes with age, with some of the most pronounced changes occurring in the first three years of life followed by a more stable adult-like microbiome developing after this time-period (Palmer et al., 2007; Koenig et al., 2011; Yatsunenko et al., 2012). Examining this pediatric oncology population by age group (U3 and O3) we found increased alpha-diversity in O3 compared to U3 population, a pattern similar to that found previously (Palmer et al., 2007; Koenig et al., 2011; Yatsunenko et al., 2012). We identified increases in *Bacteroides* and *Parabacteroides* spp in the O3 group, while *Escherichia* and *Bifidobacterium* were increased in the U3 group. The results from this patient population agree with past studies, in other populations, that show increased *Bacteroides* in the O3 and increased Enterobacteriaceae and *Bifidobacterium* (Palmer et al., 2007; Koenig et al., 2011; Yatsunenko et al., 2012) as well as Veillonellaceae in U3 (Yao et al., 2021). However, we did not find increased Firmicutes taxa in O3 in this population, which has been reported in some previous studies (Palmer et al., 2007; Yatsunenko et al., 2012). These observed taxonomic differences between the U3 and O3 populations is consistent with the differences in pathway relative abundance we observed with age. Our unique result for this patient population could be due to antibiotic use within this population, as we found antibiotics to have the largest impact on Firmicutes taxa. It is noteworthy that most studies exclude samples with antibiotic use when examining changes in the GIT microbiome. This is not possible, nor desirable, within this patient population because antibiotic use is prevalent and understanding its role has implications for management of hematologic cancer patients.

The antibiotics most used in these patients were piperacillin-tazobactam and vancomycin. The first has broad anti-bacterial activity impacting both Gram positive and Gram negative bacteria and so is expected to have large effects on the distribution of phyla. In contrast, vancomycin acts against Gram positive bacteria and so is expected to have a large effect on taxa within Firmicutes and Actinobacteria. We found that the marginal effect of antibiotic use is associated with a significant decrease in the relative abundance of Firmicutes, mostly within the Lachnospiraceae and Ruminococcaceae. Many of the Firmicutes with reduced relative abundance are important for SCFA production (*Anaerostipes, Blautia, Dorea, Roseburia, Coprococcus, Subdoligranulum*). SCFA production is necessary for GIT epithelial health including epithelial cell energy metabolism, intestinal barrier function, and immunological homeostasis (Morrison and Preston, 2016; Parada Venegas et al., 2019; Deleu et al., 2021). Interestingly, antifungal use was associated with reduced relative abundance of many of the same taxa, as well as another important lineage of SCFA producers, *Faecalibacterium*. Declines in these taxa had been previously identified as differing between pediatric ALL patients and healthy controls, and was attributed to chemotherapy treatment (Rajagopala et al., 2016). Our results suggest that antibiotic or antifungal treatment also impacts SCFA producing bacteria. It was clear from the multivariate analysis that taxa linked with SCFA production were highly impacted by both antibiotic and antifungal exposure. This finding has important implications for protective GIT barrier function. Of the SCFAs, butyrate is the main effector molecule on physiological regulation of the host GIT; it serves as an energy source for mucosal epithelial cells and it is an important regulator of inflammation, differentiation, and apoptosis in host cells (Scheppach and Weiler, 2004; Hamer et al., 2007; Pajak et al., 2007; Mowat and Agace, 2014). Butyrate affects the colonic cells, and thus gastrointestinal integrity, by inhibiting inflammation and carcinogenesis, reinforcing colonic defense, and decreasing oxidative stress *via* inhibition of nuclear factor kappa β activation and histone deacetylation (Hamer et al., 2007). Note that this patient population is subject to increased oxidative stress, and it would benefit from inhibition of inflammation and reinforced colonic defense. However, increased antibiotic and antifungal use in this same population is associated with a stool microbiome community having significantly lower relative abundance of taxa responsible for beneficial butyrate production. While the stool microbiome community does not fully represent the taxonomic complexity of the colonic luminal environment it is suggestive of changes that could impact colonic healthy. Whether patient GIT health could be improved by increasing butyrate producers (thereby improving colonic defense and gastrointestinal inflammation) without a negative impact on this already immune compromised group requires further investigation.

Also associated with antibiotic and antifungal use was a significant increase in the relative abundance of taxa present in the stool samples that are normally associated with the oral microbiome community, including *Actinomyces, Rothia, Abiotrophia*, and *Streptococcus* species. Increases in oral bacteria within the GIT microbiome have been shown in colorectal cancer patients, and this has been implicated in its pathogenesis based on their absence in healthy controls (Drewes et al., 2017; Segata, 2018; Cheng et al., 2019; Thomas et al., 2019). We suggest a possible alternative hypothesis for increased oral bacterial presence. Antacid and proton pump inhibitor use is high within our patient population, and we hypothesize that their use promotes oral bacteria to transit the stomach into the intestine. With a reduced bacterial population from chemotherapy, along with antibiotic, and or antifungal use, these oral bacteria can more readily colonize the available GIT niches. Studies examining the impact of antacids and proton pump inhibitors on microbial taxa passing from the stomach into the GIT clearly show that gastric acid reduction alters intestinal bacteria (Williams and McColl, 2006; Kanno et al., 2009; Lombardo et al., 2010; Garcia-Mazcorro et al., 2012; Freedberg et al., 2014). Studies found increases in oropharyngeal *Lactobacillus* and *Veillonella* taxa (Kanno et al., 2009) as well as decreases in Bacteroidetes (Freedberg et al., 2014). In addition, enteric infection and bacterial overgrowth have been related to gastric acid reduction (Drasar et al., 1969; Howden and Hunt, 1987; Thorens et al., 1996; Husebye, 2005; Freedberg et al., 2014). Oncology patients frequently use antacids as a result of chemotherapy treatment, however the impact of acid reduction on the microbial landscape concurrent with other sources of GIT dysbiosis has yet to be examined.

Fungal infections can occur in oncology patients due to decreased immunity and often require the use of systemic antifungal agents. While many studies have examined the impact of antibiotic use on the GIT microbiome far fewer have examined antifungal use on the microbiome, with most using mouse models (Qiu et al., 2015; Sam et al., 2017; Udawatte et al., 2020; Heng et al., 2021). Past mouse studies looking at antifungal use identified *Bacteroides*, *Alistipes*, *Lactobacillus*, some Firmicutes, and Proteobacteria taxa to be increased while members of the Clostridium XIVa were decreased (Qiu et al., 2015; Udawatte et al., 2020; Heng et al., 2021). We however identified significantly decreased Bacteroidetes taxa (*Bacteroides*, *Parabacteroides*, and *Alistipes*) as well as Lachnospiraceae, and Ruminococcaceae, including butyrogenic (*Faecalibacterium, Subdoligranulum*, and *Anaerostipes*), and acetogenic taxa (*Blautia*) among patients treated with antifungals. We also identified significantly increased lactic acid bacteria (*Enterococcus*, and *Streptococcus*), Actinobacteria, and several potential opportunistic pathogens from the Proteobacteria (*Klebsiella*, *Haemophilus*, and *Enterobacter*), and Firmicutes (*C. paraputrificum*, and *Clostridioides difficile*) among samples treated with antifungals. The microbial community we observed in patients treated with antifungals closely reflects that reported as associated with *C. difficile* infections; i.e., decreased diversity, scarcity of Firmicutes, decreased relative abundance of Ruminococcaceae, and Lachnospiraceae, which results in low levels of butyrogenic and acetogenic taxa and increased relative abundance of lactic acid bacteria (Antharam et al., 2013; Vakili et al., 2020). These findings suggest that antifungal use in our patient population may contribute to an environment favorable to *C. difficile*. Even if patients receiving antifungals already begin treatment with more opportunistic pathogens such as *C. difficile*, managing the effect of antifungal treatment on the host microbiome could have positive effects on patient outcomes. Future investigation of the relationship between the use of antifungals, their effect on the commensal microbiome, and the distribution of *C. difficile* prior to, and throughout, patient care is warranted.

The clinical care for leukemia and lymphoma patients, despite the relative rarity of active fungal disease, sometimes includes prophylactic antifungal treatment. We found that antifungal treatments had profound effects on bacterial community composition and, surprisingly, was the single biggest factor affecting the functional capacity of the microbial community. Furthermore, the majority of the microbial pathways affected by antifungal treatment were associated with bacterial metabolic activities. In the context of ecological coexistence theory (Chesson, 2000), these findings raise the possibility that antifungal treatments are having profound and unexpected effects on bacterial community ecology. A core concept of community ecology is that direct competition between species leads to competitive exclusion, and a loss of diversity (Hardin, 1960). Because community-level metabolic activities mediate consumer–resource interactions among GIT microbes, ecological coexistence theory predicts that community diversity can be maintained *via* metabolic niche differentiation (Johnson and Bronstein, 2019; Johnson, 2021). From the ecological perspective, metabolic resource partitioning within the human GIT should therefore discourage interspecific competition and serve as a stabilizing mechanism for the GIT community. The observation that antifungal treatment has profound effects on both species composition and functional capacity implies that fungal metabolic activities play a critical role in resource partitioning. A possible mechanism is fungal degradation of carbohydrates that are otherwise inaccessible to fermenting bacteria (Li et al., 2020; Luo et al., 2021), which could diversify resources and thereby allow a larger fraction of the bacterial species to co-exist in the human gut. Notwithstanding the mechanistic details, altering fungal biodiversity appears to change the competitive and mutualistic dynamics among bacteria, thereby causing widespread competitive exclusion and general reductions in diversity within the human gut. Recent advances in coexistence theory are raising awareness of how competitive trade-offs impacted by antibiotic treatments could affect the persistence of pathogens in a clinical setting (Letten et al., 2021). Since very little is known about how antifungal treatment alters niche overlap in the GIT community, and the extent to which this drives changes in both commensals and drug-resistant pathogens, coexistence theory should be further developed to include antifungal treatments in a clinical setting.

While our findings have a variety of implications for both human health and microbial community stability, there are several limitations that could impact our ability to generalize our findings. While we had very strong sampling among patients will ALL, samples from AML, HL and NHL patients were more limited and so additional sampling from this population would be valuable to confirm these findings within those patient populations. In addition, collection time points between patients varied and future work should include a more balanced sampling of patients at different times of treatment. This will improve the inference of both taxonomic and functional changes that occur over the course of treatment. Lastly, empirical quantification of competitive and mutualistic coexistence of microbes within the GIT is challenging. Additional theoretical developments will be required to guide future empirical studies and, ultimately, to rigorously frame and evaluate predictions relevant to the clinical setting.

## Conclusion

Antibiotic and antifungal use are critical in the care of leukemia and lymphoma patients to prevent infections and febrile neutropenia events that would be life threatening given the decreased immunity of this cohort. We show however that the use of these compounds does impact the microbiome both taxonomically and functionally and may further contribute to a dysfunctional GIT barrier. Because shifts in microbial composition and functional pathways can cause changes in host physiology that may have a long lasting impact on future health, it is especially important to consider the effect of age. Children under 3 present a dilemma as they are more susceptible to severe infection due to their age, but also have less biodiversity in their microbiome and as such are at increased risk of diversity loss with antibiotic/antifungal use. While this is a topic of ongoing clinical investigation for safety and efficacy, it may be prudent to increase nutritional supplementation of healthy bacteria in this age group. In addition, when prophylactic acid suppression is prescribed in children with leukemia to mitigate gastric erosion caused by corticosteroids, the accompanying antacids should be discontinued as soon as the child is not receiving steroids. Lastly, the use of antifungals has received little to no attention in this vulnerable group, and there could be a larger impact on the microbiome than appreciated by clinicians. We suggest that concerted reassessment of how and when antifungals are used in this patient population is warranted with further studies in this area.

## Data availability statement

The data presented in this study are deposited in the European Nucleotide Archive (https://www.ebi.ac.uk/ena) under accession numbers: PRJEB29237, PRJEB41463, PRJEB46214, and PRJEB53954.

## Ethics statement

The study was reviewed and approved by the IWK Health Center Research Ethics Board (REB# 1019670 & 1022029). Written informed consent to participate in the study was provided by the participants’ legal guardian.

## Author contributions

TM, KK and KD contributed to the conception and design of the study and interpretation of the results. KD performed analyses and wrote the manuscript. TM, GR and JB wrote sections of the manuscript. ZF organized and maintained the patient database. KK, TM, JB, ML and JL secured funding for the project. All authors contributed to manuscript revision, read, and approved the submitted version.

## Funding

This research was funded by a Nova Scotia Health Research Foundation (now Research Nova Scotia) establishment grant, Beatrice Hunter Health Research Institute New Investigator grant, and JD Irving Foundation grant to KK. In addition, KD was funded by a IWK Research Associateship and The Weston Foundation.

## Acknowledgments

The authors would like to thank participating children and their families, and the nurses at the IWK Health Centre for assistance with sample collection.

## Conflict of interest

The authors declare that the research was conducted in the absence of any commercial or financial relationships that could be construed as a potential conflict of interest.

## Publisher’s note

All claims expressed in this article are solely those of the authors and do not necessarily represent those of their affiliated organizations, or those of the publisher, the editors and the reviewers. Any product that may be evaluated in this article, or claim that may be made by its manufacturer, is not guaranteed or endorsed by the publisher.
