# Supplementary figures and images for: Antibiotic and antifungal use in pediatric leukemia and lymphoma patients are associated with increasing opportunistic pathogens and decreasing bacteria responsible for activities that enhance colonic defense
Source: Front Cell Infect Microbiol. 2022 Jul 27;12:924707. doi: 10.3389/fcimb.2022.924707 (PMC9363618; doi:10.3389/fcimb.2022.924707)

Figure S1

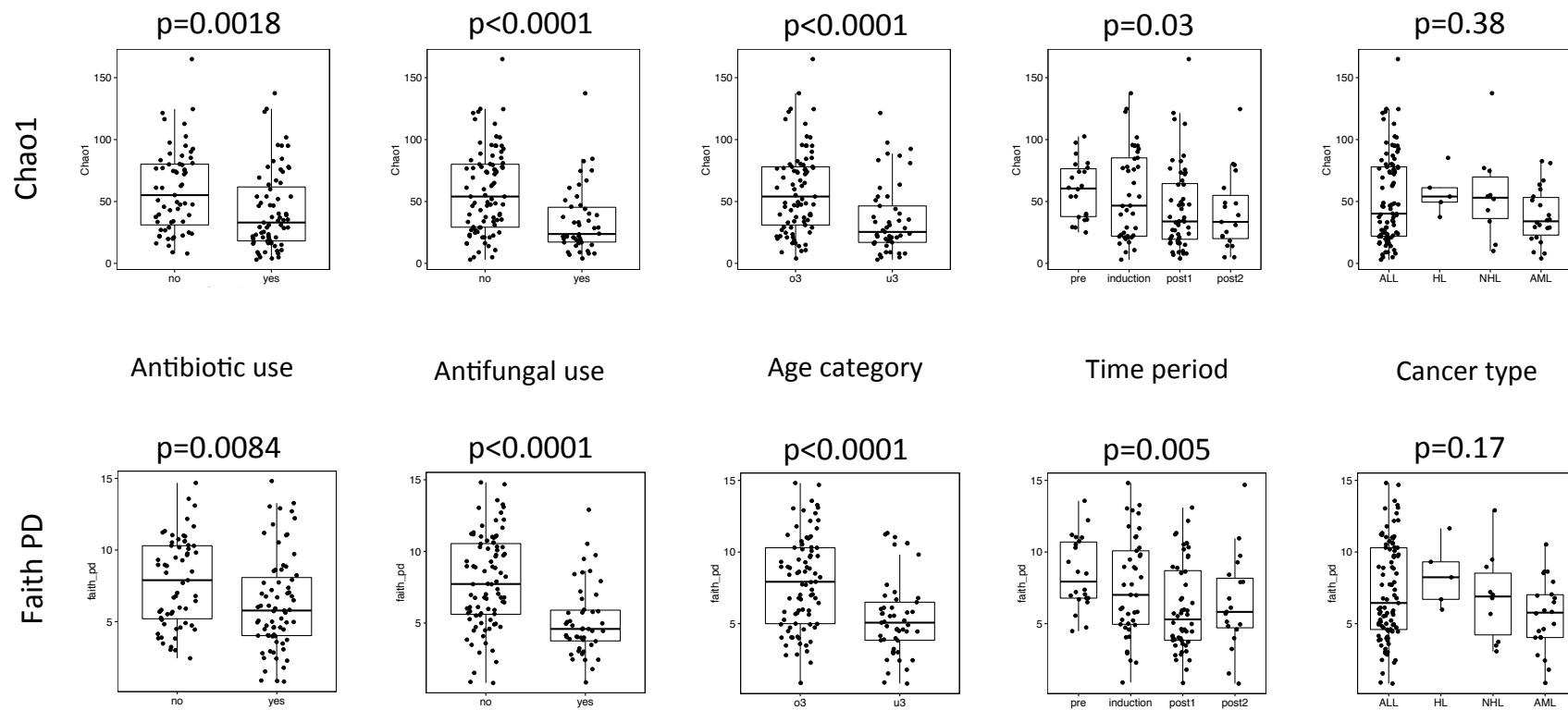

Figure S2

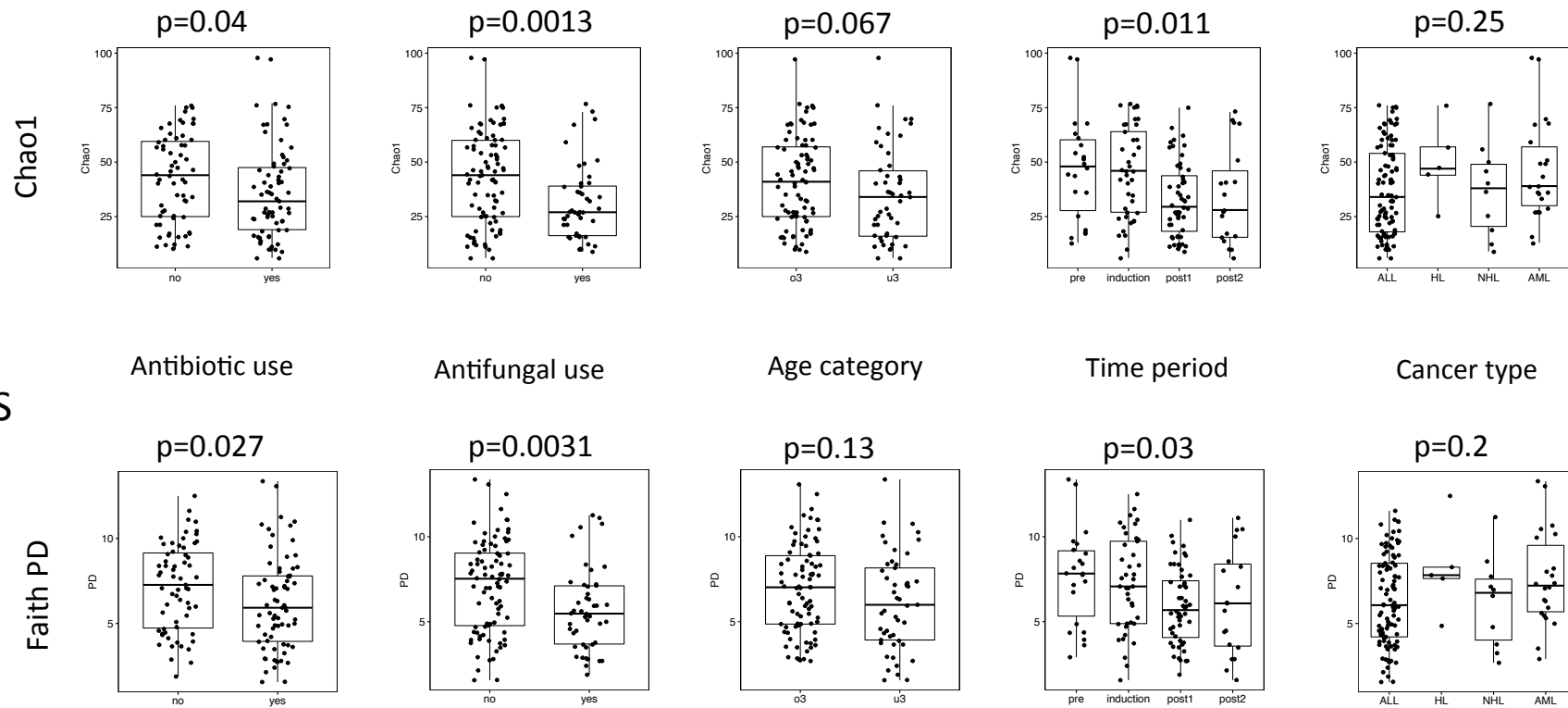

Supplement: Supplementary file 1 [file DataSheet_1.pdf]
